# Two novel real-time PCR assays for *Brucella* detection: a species-specific multiplex and a genus-level singleplex developed via large-scale whole genome sequencing data analysis

**DOI:** 10.1128/spectrum.02974-25

**Published:** 2026-03-26

**Authors:** Emily Hoover, John Chmara, Marc-Olivier Duceppe, Philippe Charron, Dara Lloyd, Hongsheng Huang, Om Surujballi, Niroshan Thanthrige-Don, Kristin Arnold, Jennifer Hazelwood, Mingsong Kang

**Affiliations:** 1 Ottawa Laboratory-Fallowfield, Canadian Food Inspection Agency 5737, Nepean, Ontario, Canada; Taichung Veterans General Hospital, Taichung, Taiwan

**Keywords:** qPCR, *Brucella abortus*, *Brucella melitensis*, *Brucella suis*, genomic data analysis, unique core sequences

## Abstract

Brucellosis, a reportable zoonosis with significant global economic and public health challenges, is primarily caused by *Brucella melitensis*, *Brucella abortus*, and *Brucella suis* in small ruminants, cattle, and swine, respectively. In addition to traditional serological tests and bacterial culture, the emergence of molecular tests, particularly quantitative PCR (qPCR), has gained attention for their rapid turnaround time, as well as their high sensitivity and specificity. However, validating existing assays using a sufficiently large and diverse data set remains challenging, and there is a pressing need for a reliable multiplex assay capable of simultaneously identifying *B. abortus*, *B. melitensis*, and *B. suis*. This study reports the development and evaluation of a highly sensitive and specific multiplex qPCR assay for detecting three *Brucella* species, alongside a novel genus-specific *Brucella* qPCR assay, both designed through large-scale comparative analysis of whole-genome sequencing (WGS) data. *In silico* validation of these primer-probe sets demonstrated sensitivities and specificities ranging from 99.8% to 100% using a large WGS data set, indicating comparable or improved *in silico* performance relative to currently published qPCR assays. Subsequently, the qPCR assays were evaluated using a comprehensive genomic DNA panel comprising 235 classical *Brucella* strains and 192 non-classical *Brucella* or non-*Brucella* samples, achieving 100% sensitivity and specificity with excellent low-level detection capability. Furthermore, these assays successfully detected *Brucella* DNA in tissue samples, demonstrating exceptional sensitivity and diagnostic accuracy through an advanced bacterial DNA enrichment process. These qPCR assays represent a valuable tool for rapid, accurate diagnosis of *Brucella* species in clinical specimens and bacterial cultures.

**IMPORTANCE:**

Brucellosis is a disease affecting animals and humans, causing major health and economic problems worldwide. It is mainly caused by *Brucella abortus*, *Brucella melitensis*, and *Brucella suis*. Quick and accurate identification of these bacteria is critical to control the spread of infection and protect public health. Current laboratory tests have limited ability to detect the previously mentioned *Brucella* species simultaneously and often lack validation across large and diverse sample sets, indicating a need for enhanced diagnostic tools. This study presents the development and evaluation of two rapid, accurate molecular tests that can detect the *Brucella* genus or precisely identify these three critical species simultaneously, thereby saving time and resources. With their excellent performance on large data sets across both computer-based and laboratory environments, these tests offer hope for improved diagnosis in both clinical and veterinary applications, thereby supporting public health and animal disease control efforts.

## INTRODUCTION

Brucellosis, a zoonotic disease caused by several species of the *Brucella* genus, remains a global issue that seriously endangers several mammalian species and poses threats to the economy and public health worldwide (1–3). Based on phylogenetic analysis, the genus *Brucella* comprises 10 classical species: *Brucella melitensis*, *Brucella abortus*, *Brucella suis*, *Brucella canis*, *Brucella neotomae*, *Brucella ovis*, *Brucella microti*, *Brucella ceti*, *Brucella pinnipedialis*, and *Brucella papionis*, along with several newly identified species (4–7). Additionally, species of *Ochrobactrum* have recently been reclassified into the *Brucella* genus, which may lead to confusion in clinical diagnostics and treatment, as *Brucella* (*Ochrobactrum*) species differ significantly from classical *Brucella* species in virulence mechanisms, infection dynamics, diagnostic profiles, and treatment strategies (8). Human brucellosis is primarily caused by three organisms, *B. abortus, B. melitensis,* and *B. suis*, which are transmitted from infected animals to humans (9, 10). Bovine brucellosis, caused by *B. abortus*, predominantly affects cattle, bison, and elk. Porcine brucellosis, resulting from *B. suis*, primarily impacts swine. Caprine/ovine brucellosis, associated with *B. melitensis*, mainly affects goats and sheep. These types of brucellosis are listed as notifiable diseases by the World Organization for Animal Health (WOAH), due to their zoonotic potential and their significant impact on international trade and public health measures (11, 12).

While brucellosis has been eradicated from livestock in several developed countries, including Canada, it continues to pose significant public health challenges in many developing and developed nations (13). Furthermore, Canada hosts two established wildlife reservoirs of brucellosis: one situated near Wood Buffalo National Park and another extending across the Arctic and sub-Arctic regions (14–19). Therefore, international trade activities or interactions with wildlife near reservoir areas pose a significant risk of reintroducing brucellosis agents into commercial livestock populations. Such reintroductions could compromise the health of livestock and humans in Canada, potentially resulting in substantial economic losses due to decreased productivity, trade restrictions, and increased public health expenditures. Additionally, it has been shown that these new *Brucella* species exhibit significant genetic diversity and flexibility, indicating high capacity for adaptation to new hosts or environments (20–22). In conjunction with various factors, including climate change and alterations in human demographic patterns, brucellosis possesses the potential to re-emerge as a significant public health concern (23).

The diagnosis of brucellosis is performed worldwide through various methods, including serological tests, bacterial culture, and molecular assays (24). Recent advancements in molecular techniques, such as polymerase chain reaction (PCR) and quantitative PCR (qPCR), have demonstrated superior specificity to serological tests and enhanced sensitivity compared to traditional bacterial culture methods, followed by conventional identification techniques, including biochemical tests (24–26). qPCR assays are increasingly favored over conventional PCR due to their rapid turnaround time, higher sensitivity, quantifiability, and objective interpretation (27, 28).

Developing species-specific qPCR assays for *Brucella* is particularly challenging due to the high genetic homogeneity among *Brucella* species. Nevertheless, several research groups have successfully designed assays targeting conserved genomic regions, although many of these efforts have relied primarily on limited genomic data sets (see Table 2). Crucially, these existing *Brucella* qPCR methods have not undergone extensive or comprehensive evaluation using large and diverse clinical or field sample sets. Additionally, these assays typically depend on BLAST-based *in silico* evaluation of individual primers and probes against public databases, most often using non-*Brucella* sequences to assess specificity, with limited and unsystematic evaluation of inclusivity across the full diversity of *Brucella* genomes. While the NCBI Basic Local Alignment Search Tool (BLAST) web interface searches provide valuable information, this approach presents several limitations, particularly for evaluating *in silico* analytical sensitivity. The tool assesses each oligonucleotide individually and does not automatically consider correct orientation, amplicon length, mismatch effects, and the probe-binding site within the predicted amplicon. Additionally, sensitivity assessment is frequently limited to a small subset of sequences unless algorithm parameters are adjusted, as the default settings return only 100 alignments. Furthermore, BLAST-based evaluation does not yield quantitative estimates of *in silico* inclusivity and exclusivity. Most importantly, public whole-genome assembly databases, such as GenBank and RefSeq, often contain misidentified, misannotated, or contaminated genome assemblies (29–31), making the taxonomic identities associated with BLAST hits unreliable. These limitations collectively hinder rigorous *in silico* assessment of the robustness and reliability of existing qPCR assays across diverse genetic backgrounds.

Additionally, a rapid, sensitive, and specific multiplex qPCR assay capable of simultaneously detecting and distinguishing *B. abortus*, *B. melitensis*, and *B. suis* is urgently needed, as no such standardized and evaluated test currently exists to address this critical diagnostic gap in both animal and human health. Such an assay would provide a robust platform for the simultaneous detection and differentiation of multiple critical *Brucella* species within a single reaction, thereby reducing assay time, reagent consumption, and labor. This streamlined workflow would be particularly advantageous for large-scale surveillance and rapid outbreak response, ultimately enhancing disease monitoring and control across veterinary and public health.

In this study, we performed a comprehensive comparative analysis of *Brucella* whole-genome sequencing (WGS) data using a *k-mer* approach to identify unique core sequences (UCSs) specific to the *Brucella* genus, as well as to the species *B. melitensis*, *B. abortus*, and *B. suis*. These UCSs were utilized to develop primers and probes for two novel qPCR assays: one targeting *Brucella* species at the genus level and a multiplex assay designed to simultaneously detect and differentiate *B. melitensis*, *B. abortus*, and *B. suis*. The initial development of these assays involved *in silico* PCR analysis using a curated and validated panel of *Brucella* and non-*Brucella* genome assemblies. This approach enforced correct orientation, predefined amplicon length, mismatch thresholds, and verification of the probe-binding site. Subsequently, these qPCR assays were evaluated through wet-lab experiments, which demonstrated exceptionally high sensitivity and specificity.

## MATERIALS AND METHODS

### Bacterial strains, culture conditions, and DNA extraction

A collection of 235 *Brucella* clinical isolates and reference strains (Table S1) is maintained at the BSL-3 laboratory at the Canadian Food Inspection Agency-Fallowfield lab (CFIA-OLF). These clinical isolates can be traced back to the 1980s, and the majority are Canadian isolates with a broad geographic distribution. These *Brucella* strains were cultured on serum dextrose agar plates and incubated at 37°C with 10% CO_2_ for 72 h. Then, the bacterial cells were washed and resuspended in physiological saline, followed by heat killing at 80°C for 20 min. Genomic DNA of all the *Brucella* isolates was extracted from heat-killed bacterial suspensions using a PureLink Genomic DNA Mini Kit (Thermo Fisher, USA), according to the manufacturer’s instructions. Extracted DNA was used to prepare Illumina sequencing libraries with the Illumina DNA Prep Kit (Illumina, USA) and sequenced on the MiSeq platform with the MiSeq reagent kit v3 (Illumina, USA). The identity of strains was confirmed from WGS data using the vSNP v2.03 pipeline (https://github.com/USDA-VS/vSNP). Additionally, genomic DNA was collected from a *Brucella* (*Ochrobactrum*) strain and 191 non-*Brucella* strains, representing approximately 16 genera (Table S1). Most of these genera are known to either cross-react in *Brucella* serological tests or commonly co-exist in clinical settings or as potential environmental contaminants. The samples were obtained from various laboratories within the Agency. Most of these clinical and environmental isolates were identified using appropriate API biochemical tests (bioMérieux Canada Inc.). Some of the clinical isolates provided have been confirmed with molecular assays, including WGS. ATCC reference strains were obtained from the American Type Culture Collection (ATCC, USA). All the bacterial DNA of these non-*Brucella* stains was extracted using PureLink Genomic DNA Mini Kit (Thermo Fisher, USA) or DNeasy Blood & Tissue Kit (Qiagen, USA). The quality of genomic DNA was evaluated by Nanodrop (Thermo Fisher, USA), and the concentration was determined by a Qubit 4 fluorometer (Thermo Fisher, USA).

### *Brucella* cell count

The total number of heat-killed bacterial cells was determined using the QUANTOM Tx Microbial Cell Counter (Logos Biosystems, Anyang-si, Gyeonggi-do, South Korea) following previously published methods (32). Briefly, 10 µL of heat-killed *Brucella* strain suspension at approximate concentrations of 2 × 10^6^ to 5 × 10^8^ colony forming unit (CFU)/mL in a sterile microfuge tube was mixed with 1 µL QUANTOM Cell Staining Dye (Cat. # Q13101) and 1 µL QUANTOM Total Cell Staining Enhancer (Cat. # Q13002) followed by brief vortexing and incubation at 37°C for 30 min in the dark. After mixing again, 8 µL of QUANTOM Cell Loading Buffer I (Cat. # Q13001) was added and mixed gently to avoid creating bubbles. Then, 5.5 µL of the stained cells was loaded into the side of a QUANTOM M50 Cell Counting slide chamber (Cat. # Q12001), followed by centrifugation of the slide at 300 RCF for 10 min. The slide was loaded into the slide port of the QUANTOM Tx Microbial Cell Counter, and each sample was read using default counting settings.

### Establishment of *Brucella* WGS data used for primers/probes design

Raw sequencing reads of *Brucella* species were acquired from the Sequence Read Archive (SRA) in October 2020. Whole genome assemblies were generated using SKESA version 2.4.0 (33) and subsequently annotated with Prokka version 1.14.5 (34). Species identification was further validated through a Mash-based phylogenetic analysis conducted using an in-house pipeline, genome_comparator version 0.1 (https://github.com/duceppemo/genome_comparator). The resulting data set consists of 1,597 *Brucella* genome assemblies, including 737 *B. abortus*, 108 *B. canis*, 59 *B. ceti*, 3 *B. microti*, 6 *B. neotomae*, 36 *B. ovis*, 12 *B. pinnipedialis*, 235 *B. suis*, 391 *B. melitensis*, and 10 genomes of unidentified *Brucella* species.

### PubMed search strategy for novel *Brucella* TaqMan-based qPCR assays

A comprehensive PubMed search was conducted for articles published from 1 January 1990 to 30 November 2025. The search required that the article title contain “*Brucella*” or “brucellosis” and that at least one of the following terms also appear in the title: “real-time,” “real time,” “qPCR,” or “real-time polymerase chain reaction.” In total, 80 articles were retrieved, of which 46 were related to TaqMan-based qPCR assays (File S2).

### Primers/probes design and *in silico* evaluation

Using the previously mentioned database, the pipeline selective_primer_finder version 0.2.1 (https://github.com/duceppemo/selective_primer_finder) and RUCS version 1.0.3 (35) were used to identify unique core sequences (UCSs) for all the classical *Brucella* species, *B. abortus, B. melitensis,* and *B. suis,* respectively, followed by primer/probes design. Multiple sets of primers and probes were designed to specifically amplify an amplicon around 100 base pairs in size.

To visualize the genomic distribution of UCSs, BLASTn-aligned positions were binned into 10 kilobase (kb) non-overlapping windows across reference genomes, including *B. melitensis* (GCF_000007125.1), *B. suis* (GCF_000007505.1), and *B. abortus* (GCF_000369945.1). The number of UCS falling within each 10 kb non-overlapping bin was calculated, and these values were used to generate a density histogram using circos v 0.69-8 (36).

All primers and probes were further assessed using in_silico_PCR v0.5 (https://github.com/duceppemo/insilicoPCR) employing 1,160 genome assemblies of classical *Brucella* species and 194 assemblies of closely related non-*Brucella* species from the NCBI RefSeq database, all of which passed average nucleotide identity (ANI) and taxonomic validation checks, with completeness scores above 80%(37) (as of March 2025) (Table S2).

Sensitivity was defined as the proportion of true-positive samples that successfully produced the expected PCR amplicon, with the probe located within the target region. Specificity was defined as the proportion of true-negative samples that did not yield a PCR product.

The primer-probe sets with the highest sensitivity and specificity were further evaluated using PrimerSuite (January 2021) for dimer formation and multiplex PCR assays (38). Primers and probes (Table 1) were synthesized by Integrated DNA Technologies (IDT) (Newark, NJ, USA).

**TABLE 1:** Primers and probes used in this study

Format	Target	Primer or probe name	Sequence 5′−3′	Amplicon size (bp)
Singleplex	Classical *Brucella* species	Bru_genus-F	CGCGCATGATACCACTTCT	
		Bru_genus-P	FAM-AGGTGCGGTCGATAAACTTCTGGAAC-QSY	93
		Bru_genus-R	GTGTTCGCGCATGTTGAAG	
Multiplex	*B. abortus*	BA-F	CATACAACCGCCATGGGT	
		BA-P	FAM-ACAGACGGTCGGCCATTCCA-QSY	75
		BA-R	CTCTCGCGCCGTATCAG	
	*B. melitensis*	BM_10-F	ATGGTCGGCAACGAGTG	
		BM_10-P	ABY-CTGCCTGTTTGGCCTGATAGT-QSY	147
		BM_10-R	GCGGCGAAGGAACAAGT	
	*B. suis*	BS-F	CCATAAAGCGCAAAGATCACAA	
		BS-P	JUN-CACCGTAGAGAATGCCCACTGCAT-QSY	102
		BS-R	AACCCTGTCGTCATGAAGTG	

### Real-time qPCR assays

According to the primers’ melting temperatures (Tm), annealing temperatures were optimized within the range of 62°C–66°C for the multiplex detection and quantification of *B. abortus*, *B. melitensis*, *B. suis*, as well as genus-specific *Brucell*a targets, employing the ABI 7500 real-time PCR platform (Applied Biosystems, USA). The template concentration was also optimized to ensure that Ct values of the qPCR assays fell within the optimal range of 20–30. Higher input amounts were used, as they are more likely to yield reproducible Ct values and minimize technical variation (39). The standard final primer-probe concentrations (500 nM of forward and reverse primers, and 250 nM of probe) were used in all the qPCR assays. To verify whether these standard conditions are suitable for use, the standard curve, followed by qPCR efficiency analysis, was conducted.

The optimal reaction mixture components for a 20 μL reaction mixture contained 3 µL of 0.1 ng/µL template DNA, 1× TaqMan Multiplex Master Mix (Applied Biosystems, USA), 500 nM concentration of each primer, 250 nM concentration of each probe (Table 1), and 1× TaqMan exogenous IPC MIX/DNA (Applied Biosystems, USA). Amplification and real-time fluorescence detection were performed on the ABI 7500 Fast real-time PCR system (Applied Biosystems, USA) using the following protocol: 20 s at 95°C followed by 40 cycles of 95°C for 3 s and 62°C for 30 s. Each sample had two technical replicates per experiment.

In the analysis of qPCR data, the threshold line is established at a consistent value of 0.1 for all real-time reactions. This standardization facilitates comparative studies across different sample batches and multiple qPCR runs, thereby enhancing the reliability and accuracy of the results obtained.

### qPCR efficiency

Standard curves were established with 1:10 serial dilutions ranging from approximately 2 × 10^−5^ to 2 ng/µL of genomic DNA from *B. abortus*, *B. melitensis,* and *B. suis*. The efficiency of the PCR was calculated using the qPCR Efficiency Calculator on Thermo Scientific Web Tools (https://www.thermofisher.com/ca/en/home/brands/thermo-scientific/molecular-biology/molecular-biology-learning-center/molecular-biology-resource-library/thermo-scientific-web-tools/qpcr-efficiency-calculator.html) accessed in February 2021. Standard curve experiments were performed in duplicate and repeated independently on three different occasions. Additionally, new batches of primers and probes were assessed for their qPCR efficiency before utilization.

### Sensitivity, specificity, and optimal cutoff points

A receiver operating characteristic (ROC) curve analysis was performed using the R package cutpointr v 1.1.2 (https://github.com/Thie1e/cutpointr), and the results were plotted using GraphPad Prism 7 (GraphPad Software, USA). Specifically, optimal cutoff points (cutoff Ct value) were determined by maximizing the Youden Index after kernel smoothing the distributions of the two classes. Sensitivity was calculated by dividing the number of true-positive samples with a lower optimal cutoff Ct value by the total number of true-positive samples. Specificity was calculated by dividing the number of true-negative samples with a higher cutoff Ct value by the total number of true-negative samples.

### Repeatability and reproducibility evaluation of qPCR assays

To evaluate the repeatability of the qPCR assays (intra-assay variability), high- and low -concentration *Brucella* genomic DNA samples (0.1 ng/µL and 1 × 10^−4^ ng/µL), derived from ten different *Brucella* isolates, were used for each qPCR target. To assess reproducibility (inter-assay variability), 10 independent qPCR runs were conducted using both high (0.1 ng/µL) and low concentrations (1 × 10⁻⁴ ng/µL) of *Brucella* genomic DNA. Each sample was tested in duplicate within a single run. The coefficients of variation (CVs) were calculated as described previously (40).

### Determination of the real-time PCR limit of detection

The limit of detection (LOD) was determined following a previously described method (41). Briefly, 10 independent replicates for each of the ten 10-fold serial dilutions of *Brucella* genomic DNA were used as templates in qPCR assays. The resulting data were subjected to logit analysis using GraphPad Prism 7. Based on the generated concentration-response curve, the LOD was defined as the log₁₀ of copy number at which 95% of the replicates yielded a positive result. Corresponding cutoff Ct values were derived from the standard curve generated by linear regression of DNA concentration versus Ct values.

### Evaluation of qPCR performance using clinical samples

*Brucella*-positive and -negative tissue samples were maintained in the BSL-3 laboratory at CFIA-OLF (Table S3). All samples were heat-killed in a BSL-3 environment at 80°C for 20 min. Sterility testing was performed according to our standard operating procedure to confirm the absence of viable organisms. The release of *B. suis* biovar 4 DNA into the supernatant after heat treatment was monitored using a Qubit 4 fluorometer with a Qubit 1X dsDNA HS Assay Kit (Thermo Fisher, USA). Spiked *Brucella* tissue samples were prepared by adding serial 10-fold dilutions of heat-killed *Brucella suis* biovar 4 cultures to 10 mL of heat-killed *Brucella*-negative, homogenized porcine tissue (Table S3). Accordingly, this experiment was designed to assess overall workflow performance, rather than kit-specific lysis efficiency. For DNA extraction, 10 mL of each spiked tissue sample and 3 mL of either *Brucella*-negative or *Brucella*-positive homogenized clinical samples were processed using the Ultra-Deep Microbiome Kit (Molzym, Germany) following a modified protocol (42) or PureLink Genomic DNA Mini Kit (Thermo Fisher, USA). DNA quality was assessed using a NanoDrop spectrophotometer (Thermo Fisher, USA), and the concentration was measured using a Qubit 4 fluorometer (Thermo Fisher, USA). Undiluted DNA samples were used as templates to run qPCR assays as mentioned above.

## RESULTS

### Identification of unique core sequences (UCSs) for primer and probe design

**Fig 1:**
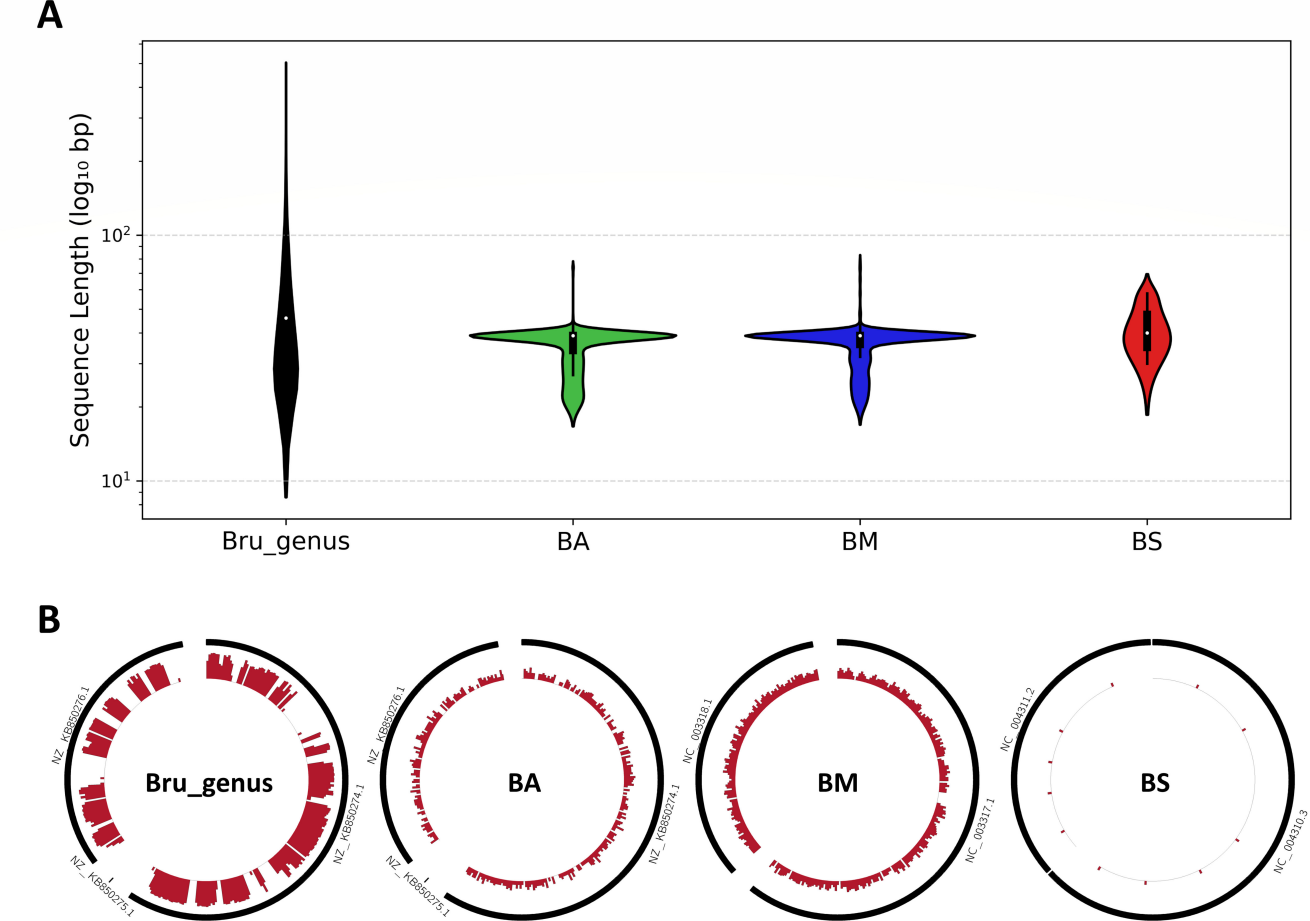
Distribution of lengths and genomic localization of unique core sequences (UCSs) in *Brucella* species. (**A**) Distribution of lengths of UCSs identified in the *Brucella* genus, *B. abortus*, *B. melitensis*, and *B. suis,* respectively. Each violin represents the length distribution of UCSs, displayed on a log_10_ scale. (**B**) Genomic distribution of UCSs across the chromosomes of *Brucella* species. BLASTn-aligned positions were binned into 10 kb non-overlapping windows, and counts were log_10_-transformed to reflect UCS density. Bru_genus: the *Brucella* genus primarily includes the classical *Brucella* species; BA: *B. abortus*; BM: *B. melitensis*; and BS: *B. suis*. The following *Brucella* reference genomes were used: *B. melitensis* (GCF_000007125.1), *B. suis* (GCF_000007505.1), and *B. abortus* (GCF_000369945.1).

Due to significant biological and genetic distinctions remaining between these *Brucella* (*Ochrobactrum*) species and classical *Brucella* species (8), in this study, we still employed *Brucella* (*Ochrobactrum*) species genome assemblies as a negative group to identify unique coding sequences (UCSs) specific to classical *Brucella* species. Through this analysis, a total of 21,678 UCSs with an average length of 59.5 bp were identified (Fig. 1A). Among these UCSs, 17,341 UCSs of classical *Brucella* species, each longer than 28 bp and distributed across both chromosomes (Fig. 1B), were subsequently used for primer and probe design. Similarly, the UCSs for *B. abortus*, *B. melitensis*, and *B. suis* were also assessed. Among these species, *B. melitensis* exhibited the highest number of UCSs with a total of 1,191 UCSs exceeding 28 bp in length, whereas *B. suis* had only 11 UCSs identified across biovars 1–4 (Fig. 1A and B). Notably, during the analysis of the genome assemblies for *B. suis* biovar 1–5, no UCSs were detected, indicating significant variations among the biovars of *B. suis*, particularly biovar 5.

Based on the identified UCSs, primer and probe sets were designed for each target, specifically focusing on the *Brucella* genus, primarily including classical *Brucella* species, *B. abortus*, *B. melitensis*, and *B. suis* (Table 1).

### *In silico* analysis of qPCR assay performance

A comprehensive evaluation of the primers and probe sets was systematically conducted using *in silico* PCR analysis, which allowed for the assessment of specificity and sensitivity. Genome assemblies of *Brucella* and closely related genera within the *Brucellaceae* family, such as *Pseudochrobactrum*, *Paenochrobactrum*, and *Falsochrobactrum*, sourced from the NCBI RefSeq database, were used to establish an *in silico* validation panel containing 1,160 classical *Brucella* isolates and 194 *Brucella* (*Ochrobactrum*) or non-*Brucella* species (Table S2).

The final selection of primers and probes (Table 1) demonstrated exceptionally high *in silico* sensitivity and specificity (Table 2). We also observed that several *Brucella* assemblies failed to yield any PCR products when utilizing primers and probes specific to the *Brucella* genus and *B. suis* (Table 2). This observation may be due to incomplete or misassembled sequences or potential sequencing errors. For instance, within the multiplex qPCR assay designed to detect *B. suis*, two assemblies (GCF_000292005.1 and GCF_000292105.1) consisted of over 250 contigs, with primers situated at the boundaries of two separate contigs. As a result, these assemblies did not produce PCR products during *in silico* PCR analysis (Table S4). Upon excluding these two fragmented assemblies, the multiplex qPCR assay demonstrated 100% sensitivity and specificity in detecting *B. abortus, B. melitensis,* and *B. suis in silico*. In the context of the *Brucella* genus qPCR assay, two genome assemblies failed to yield amplification signals: one *Brucella abortus* assembly (GCF_023651695.1) and one *Brucella melitensis* assembly (GCF_002290125.1), both of which exhibited more than three mismatches at the primer binding sites. These findings further emphasize the high efficacy of the developed primers and probes in supporting the accurate detection of *Brucella* species through *k-*mer-based methodologies.

**TABLE 2:** *In silico* analysis of TaqMan-based qPCR assays from this study and previous publications^*f*^

Target	Gene or locus tag	Amplicon size (bp)	In the previous studies or in this study		*In silico* analysis in this study		Reference
			No. of isolates *Brucella*/non-*Brucella*	No. of clinical samples	Sensitivity (%)	Specificity (%)	
*Brucella* genus^*a*^	B977_RS112010^*b*^	93	235/192	25	99.8	100	This study
	*bcsp31*	154	n.a.	487	100	100	(43)
	*bcsp31*	166	40/14	62	100	100	(44)
	*bcsp31*	55	26/68	n.a.	100	100	(45)
	*bcsp31*	165	30/8	897	99.9	100	(46)
	*bcsp31*	223	n.a	2	99.7	100	(47)
	*bcsp31*	138	n.a.	46	98.5	100	(48)
	*bcsp31*	151	25/59; 248/67	1,658	85.2	100	(49–51)
	*IS711*	128	n.a.	61	80.4	100	(52)
	*IS711*	150	13/7	178	81.0	100	(53)
	*IS711*	63	65/49	135	86.4	100	(54, 55)
	*IS711*	178	26/68	n.a.	74.3	100	(45)
	*IS6501*(*IS711*)	128	5/5	n.a	80.4	100	(56)
	*eryC*	75	177/107	n.a.	99.7	100	(57)
	16S-23S_ITS	72	7/9	56	99.7	100	(58)
	*per*	68	26/68	n.a.	99.8	100	(45)
	*eryD*	92	5/5	n.a.	99.3	60.3	(56)
	*virB4*	177	n.a.	108	89.3	100	(59)
*B. abortus*	B977_RS114980^*b*^	75	235/192	25	100	100	This study
	IS711_*alkB^c^*	136	25/59; 248/67	n.a.	58.6	100	(49, 51)
	BruAb2_0168	81	65/49	n.a.	95.8	99.9	(54)
	alkB_BruAb2_0691	174	10/28	12	54.4	100	(60)
*B. melitensis*	BME_RS11770^*b*^	147	235/192	25	100	100	This study
	IS711_BMEI1162^*c*^	279	25/59; 248/67	n.a.	92.4	100	(49, 51)
	Acetyl-CoA acetyltransferase	64	157/45	n.a.	100	100	(61)
	BMEII0466	67	65/49	31	88.6	94.0	(54)
	BMEI1162_NCR^*d*^	152	10/28	12	50.6	99.5	(60)
	IS711	251	21/12	180	90.2	100	(62)
	Omp31	121	2/3	106	99.2	29.6	(63)
*B. suis*	BR_RS17450^*b*^	102	235/192	25	100^*e*^	100	This study
	BS1330_II0657	106	100/30	n.a.	100^*e*^	100	(64)
	BR0952	83	65/49	n.a.	98.8	97.1	(54)
	BR1673(IS771)-BR1674	127	10/28	12	20.0	100	(60)

^*a*^ *Brucella* genus: primary classic *Brucella* species.

^*b*^ Reference genome assemblies were used: *B. abortus* (*B. abortus* (GCF_000369945.1), *B. melitensis* (GCF_000007125.1), and *B. suis* (GCF_000007505.1).

^*c*^ IS711 element downstream of the *alkB* gene or BMEI1162.

^*d*^ NCR: non-coding regions.

^*e*^ Two over-fragmented assemblies (GCF_000292005.1 and GCF_000292105.1) were removed from data analysis (Table S4).

^*f*^ n.a.: not applicable.

Additionally, a comparative *in silico* assessment was performed to evaluate qPCR assays developed in this study against other published *Brucella* conventional TaqMan-based qPCR assays that use a single primer-probe set per target (Table 2). Using our predefined search strategies, 29 unique qPCR primer-probe sets were identified and included in the analysis. All *Brucella* genus-specific qPCR assays evaluated demonstrated high specificity for detecting classical *Brucella* species, except for the primer-probe set targeting the *eriD* gene, which also yielded positive results in approximately 50% of *Brucella* (*Ochrobactrum*) genome assemblies. The *bcsp31* gene and IS711 remain the most widely used molecular biomarkers for primer-probe design for *Brucella* genus identification. Overall, *BCSP31*-targeting qPCR assays showed higher *in silico* sensitivity than most other targets, particularly including three primer-probe sets with perfect performance (100% sensitivity and specificity) (Table 2). The *Brucella* genus-specific assay developed in this study achieved 99.8% sensitivity and 100% specificity, placing it within a near-perfect performance group that also includes assays targeting *bcsp31*, *eryC*, *per*, and the 16S–23S rDNA internal transcribed spacer (ITS) (Table 2). Moreover, the multiplex qPCR assay developed in this study for detecting *B. abortus*, *B. melitensis*, and *B. suis* achieved 100% sensitivity and 100% specificity *in silico*, demonstrating performance equal to or superior to currently available TaqMan-based qPCR assays for these pathogens (Table 2).

### Efficiency of qPCR assays

**Fig 2:**
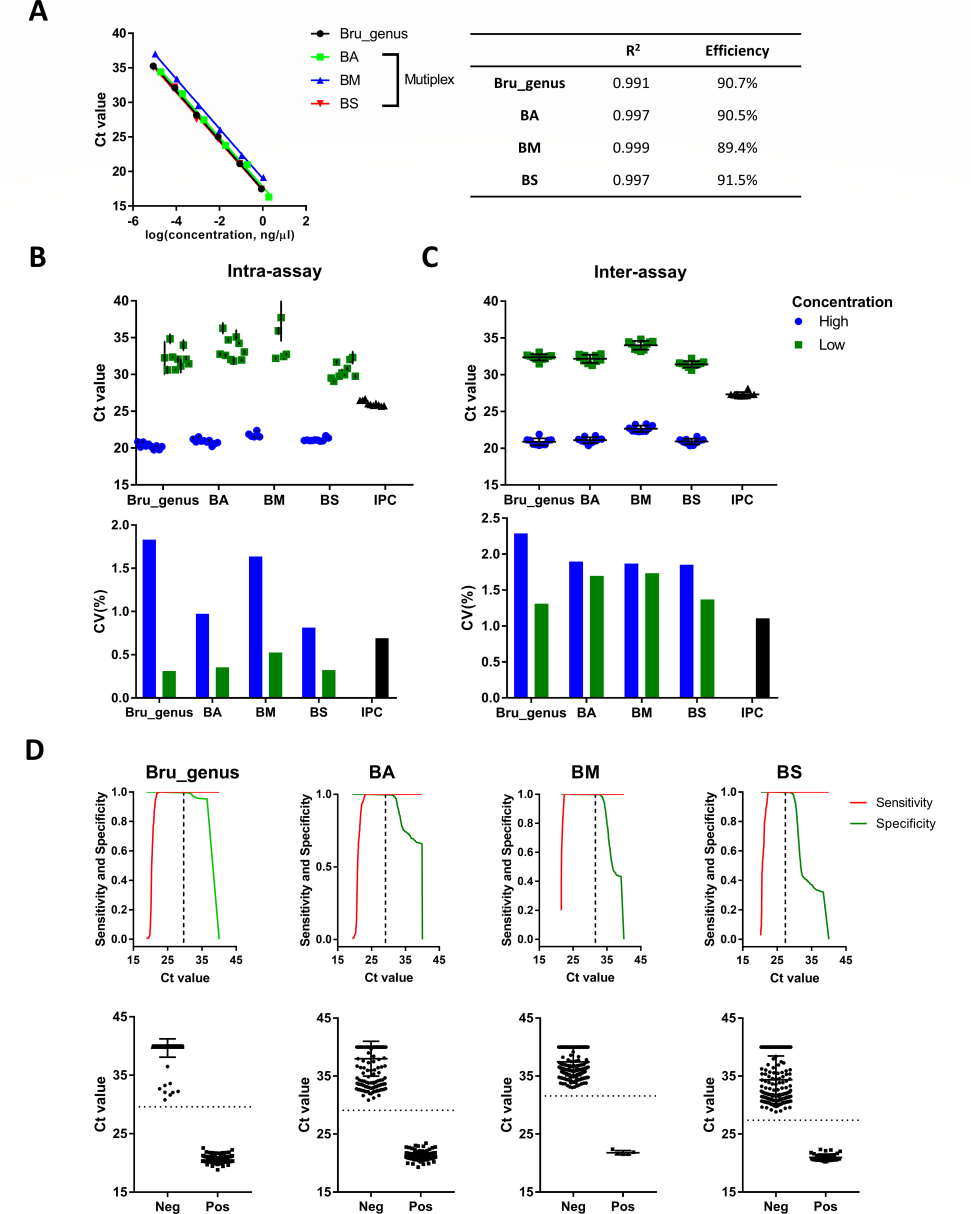
Evaluation of the performance of two novel qPCR assays developed in this study. (**A**) Standard curves of qPCR assays targeting *Brucella* genus, *B. abortus*, *B. melitensis*, and *B. suis*. *R*² and slope values were derived from linear regression analysis, and slope values were used to calculate amplification efficiency. (**B and C**) Assessment of repeatability (**B**) and reproducibility (**C**) of the assays. Repeatability was evaluated across 10 *Brucella* genomic DNA samples at high (0.1 ng/μL) and low (1 × 10^−4^ ng/μL) concentrations(top panel), with the coefficient of variation (CV) calculated per sample and summarized as a mean CV (bottom panel). Reproducibility was assessed using 10 independent replicates of the same sample for each target at high (0.1 ng/μL) and low (1 × 10^−4^ ng/μL) DNA concentrations (top panel), and CV values were calculated across replicates (bottom panel). (**D**) Receiver operating characteristic (ROC) analysis was used to determine optimal Ct cutoff values for each target, balancing sensitivity and specificity. Dashed lines indicate optional cutoffs on the sensitivity-specificity curves (top panel) and Ct distributions (bottom panel). Ct values recorded as “undetermined” in qPCR assays were set to 40 for data analysis. Bru_genus, a genus-specific *Brucella* qPCR assay targeting classical *Brucella* species; BA, BM, and BS: a multiplex qPCR assay targeting *B. abortus* (BA), *B. melitensis* (BM), and *B. suis* (BS).

Standard curves were generated for four targets: the singleplex qPCR assay for the *Brucella* genus, primarily including classical *Brucella* species, and the multiplex qPCR assay for detecting *B. abortus*, *B. melitensis*, and *B. suis*. Each assay exhibited a strong linear relationship between log-transformed genomic DNA concentrations and Ct values with R^2^ value ranging from 0.991 to 0.999 (Fig. 2A). The calculated slopes were used to estimate amplification efficiency, yielding efficiencies of over 90% for all qPCR assays except for the primers and probe used in the multiplex qPCR assay to detect *B. melitensis* (Fig. 2A). Based on the qPCR efficiency criteria previously described (41, 65), the findings suggest that the genus-specific qPCR assay is effective for both quantitative and qualitative applications, as evidenced by its amplification efficiency falling within the optimal range of 90%–110%. Conversely, the multiplex qPCR assay, which maintains efficiencies within the broader acceptable range of 80%–120%, can detect *B. abortus*, *B. melitensis*, and *B. suis*, thus making it suitable for binary evaluations.

### Repeatability and reproducibility of qPCR assays

To assess the repeatability and reproducibility of our qPCR assays, high- and low- concentration *Brucella* genomic DNA samples, derived from 10 different *Brucella* isolates for each qPCR target, were utilized in the intra-assay evaluation. For the inter-assay analysis, we performed 10 independent replicates using both high and low concentrations of *Brucella* genomic DNA. The coefficients of variation (CV) for each target in intra- and inter-assay evaluations were consistently below 2.5% (Fig. 2B and C), demonstrating the high reliability of the assay reactions.

### Evaluation of the performance of qPCR assays using bacterial genomic DNA

The developed novel qPCR assays were evaluated using a comprehensive validation panel that included genomic DNA extracted from various bacterial species (Table S1). This panel consisted of 235 classical *Brucella strains*, including 170 *B. abortus*, 38 *B. suis*, and 5 *B. melitensis*, all confirmed by WGS. In addition, one *Brucella* (*Ochrobactrum*) strain and 191 non-*Brucella* strains were included in the panel, as detailed in Table S1.

To assess the discriminatory power of the assays, an ROC analysis was performed, demonstrating a high level of performance for both qPCR assays (Fig. 2D). Both assays achieved 100% sensitivity and specificity at Ct values ranging from approximately 24 to 30 (Fig. 2D). The optimal diagnostic cutoff Ct values were identified as 29.7 for the *Brucella* genus-specific qPCR assay, and 29.1, 31.6, and 27.4 for the multiplex qPCR assay targeting *B. abortus*, *B. melitensis*, and *B. suis*, respectively. These cutoff values could effectively distinguish negative from positive samples for each assay, ensuring 100% sensitivity and 100% specificity (Fig. 2D).

### Detection of *Brucella* DNA in tissue samples

**Fig 3:**
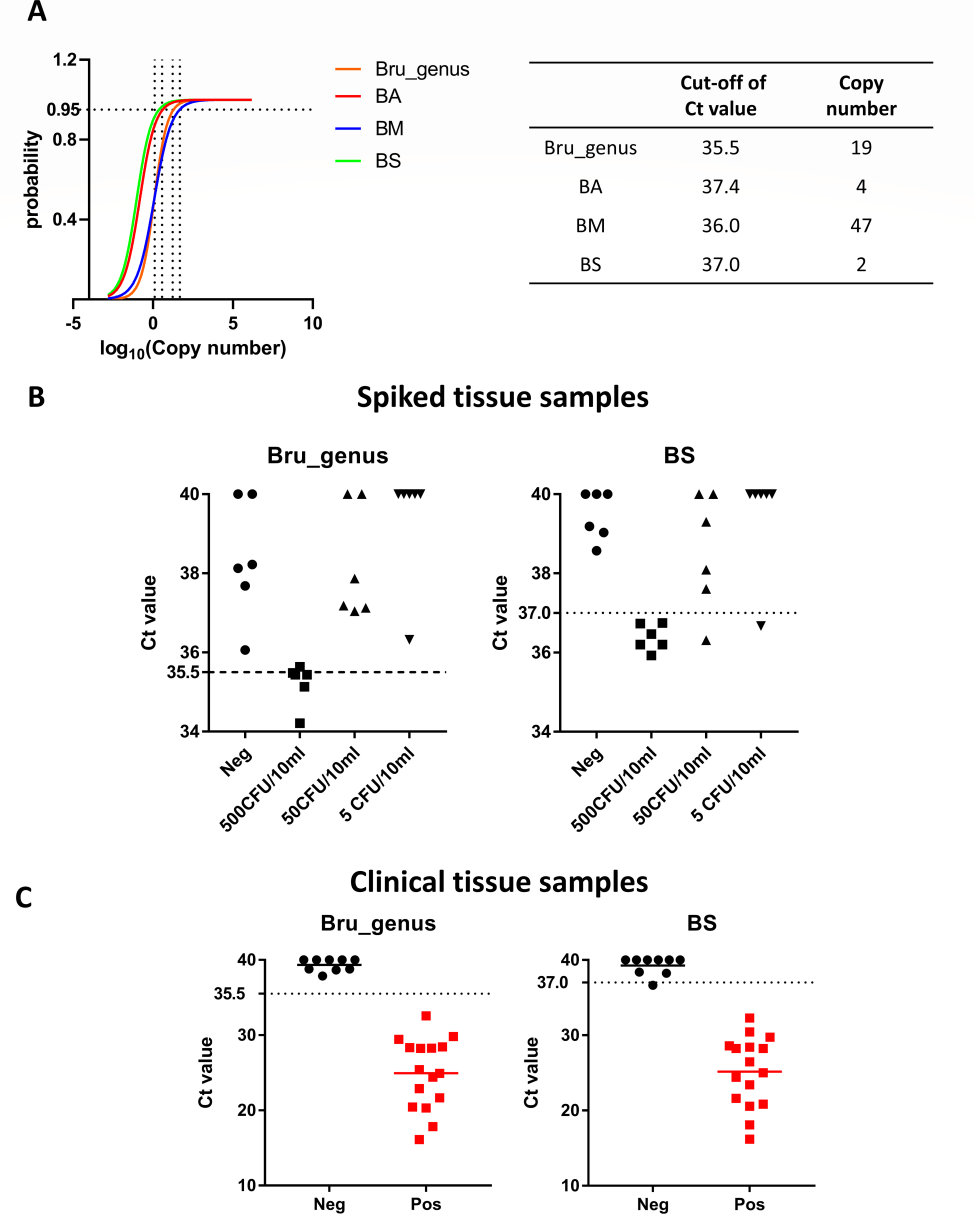
Evaluation of qPCR assay performance using spiked and clinical tissue samples prepared with the Molzym Ultra-Deep Microbiome Prep workflow. (**A**) Determination of the LOD using logit regression analysis. The LOD was defined as the DNA concentration at which 95% of replicates yielded positive qPCR results. Corresponding Ct cutoff values were derived from the standard curve generated by linear regression of log-transformed DNA concentrations versus Ct values. (**B**) Detection of *B. suis* in negative porcine tissue spiked with varying concentrations of *B. suis* bv. 4 clinical isolates, followed by extraction using the Ultra-Deep Microbiome Prep workflow. The results are shown for both the genus-level singleplex qPCR (left panel) and the *B. suis*-targeted multiplex qPCR (right panel). (**C**) Detection of *B. suis* in clinical porcine tissue samples using the same Ultra-Deep Microbiome Prep workflow. Both the genus-level singleplex (left panel) and *B. suis*-specific multiplex qPCR (right panel) were used. "Neg" indicates *Brucella*-negative porcine tissue, and "Pos" refers to culture-positive clinical samples. Ct values recorded as "Undetermined" in qPCR assays were set to 40 for data analysis. Bru_genus, a genus-specific *Brucella* qPCR assay targeting classical *Brucella* species; BA, BM, and BS: a multiplex qPCR assay targeting *B. abortus* (BA), *B. melitensis* (BM), and *B. suis* (BS).

Due to the highly variable and often unquantifiable nature of the targeted DNA extracted from clinical samples, cutoff values derived from the fixed concentration (0.1 ng/µL) of bacterial genomic DNA cannot be directly applied to complex clinical specimens. As a result, we employed a limit of detection (LOD)-based approach to define Ct cutoff values for qPCR assays detecting *Brucella* DNA in clinical specimens. LODs for each target were determined using logit regression analysis, and the concentration corresponding to a 95% detection probability was interpolated (Fig. 3A), resulting in LODs of approximately 19 genome copies for the *Brucella* genus, 4 genome copies for *B. abortus*, 47 genome copies for *B. melitensis*, and 2 genome copies for *B. suis*. These values correspond to Ct cutoff values of 35.5, 37.4, 36.0, and 37.0, respectively.

To improve the purification and enrichment of bacterial DNA from tissue matrices, a preliminary comparison was conducted between the PureLink Genomic DNA Mini Kit, which is routinely used in the laboratory, and the Molzym Ultra-Deep Microbiome Prep kit with a modified workflow designed to enrich microbial DNA from complex samples. The Ultra-Deep Microbiome Prep workflow demonstrated superior performance compared to PureLink (Fig. S1) and was therefore selected for exclusive use in all subsequent LOD experiments. Using *Brucella*-negative porcine tissue samples spiked with *B. suis* bv.4 and processed with the Ultra-Deep Microbiome Prep workflow, both genus-level simplex and *B. suis*-targeted multiplex qPCR assays could reliably detect *B. suis* at concentrations as low as 500 CFU/10 mL, achieving 83% and 100% detection probability (Fig. 3B), respectively. Notably, under the same extraction workflow, the multiplex qPCR assay was capable of detecting *B. suis* in spiked tissue at concentrations as low as 5 CFU/10 mL, highlighting its high analytical sensitivity (Fig. 3B).

Utilizing the same Ultra-Deep Microbiome Prep Kit workflow, total genomic DNA was extracted from 25 clinical tissue samples (Table S3). Among these samples, 16 were identified as *B. suis* culture-positive. Following an extensive analysis of these samples, both the *Brucella* genus-specific simplex qPCR and the multiplex qPCR specific for *B. suis* demonstrated a high efficacy in detecting *B. suis* genomic DNA across all tissue samples that tested positive for culture (Fig. 3C). Conversely, these assays produced negative results in culture-negative tissue samples, with one exception identified in the multiplex qPCR assay (Fig. 3C). This exception exhibited a CT value of 36.6, marginally below the cutoff value of 37.0. Notably, this particular sample yielded a negative result in our *Brucella* genus qPCR assay. These data suggest that both assays should be used on clinical samples to increase specificity.

## DISCUSSION

The reliability of qPCR assays highly relies on the sensitivity and specificity of primers/probes, which must be sufficiently sensitive to detect all target organisms or genes, while also being specific enough to theoretically exclude all non-targets (66, 67). This requirement poses a significant challenge when working with classical *Brucella* species, especially with *B. abortus*, *B. melitensis*, and *B. suis*, which share over 97% genomic similarity, with numerous genes or loci exhibiting nucleotide identity ranging from 98% to 100% (4, 68). This close genetic relationship complicates the identification of unique and sensitive molecular biomarkers essential for designing primers and probes capable of reliably distinguishing between the different species.

To overcome these challenges, in this study, we employed a sophisticated *k-mer*-based approach that systematically scans whole genome sequences to identify UCSs specific to the classical *Brucella* species as well as its reportable species, *B. abortus*, *B. melitensis*, and *B. suis*. This modern approach is unbiased and avoids the need for sequence alignment of conserved genomic regions, as required by traditional biomarker discovery methods. Consequently, it enables highly efficient primer design, particularly when working with large volumes of WGS data. Focusing on these key aspects enhances the likelihood of developing distinct primers and probes essential for accurate pathogen detection. The increasing adoption of this methodology reflects its growing importance in molecular diagnostics, particularly in improving the specificity and efficiency of pathogen identification (69–71).

Nevertheless, this approach necessitates using highly accurate sequencing data and precise taxonomy to ensure the validity of genome assemblies for both positive and negative groups. Consequently, before identifying UCSs for *Brucella* species, we conducted a comprehensive re-evaluation of the taxonomy of the *Brucella* genome assemblies used in this study and discovered that several published *Brucella* assemblies, despite having passed ANI and taxonomic validation checks on the NCBI website, were incorrectly identified at the species level. This misclassification was substantiated by phylogenetic analyses utilizing Mash distance and single-nucleotide polymorphism (SNP) data, as well as corroborating findings from PubMLST (72) (Table S5 and Fig. S2). However, GTDB-TK (73), a bioinformatic tool that uses ANI value for genome classification and species assignment, struggled to distinguish between *Brucella* species with its default settings (Table S1). Furthermore, a comprehensive ANI analysis revealed that solely relying on ANI presents challenges in distinguishing between *Brucella* species (Fig. S3), a conclusion consistent with prior studies (29, 68). These findings suggest that, in addition to ANI values, supplementary methodologies, such as phylogenetic analysis and PubMLST (72), are essential for the accurate identification of *Brucella* species due to their high genomic similarity.

A comprehensive evaluation and validation of qPCR assays requires extensive testing of a wide range of clinical samples or bacterial isolates to ensure accuracy, reproducibility, and diagnostic reliability. However, acquiring a sufficiently large and varied set of clinical specimens or bacterial strains often poses a significant obstacle in developing and evaluating assays. This challenge is especially pronounced in the context of rare or regulated pathogens and resource-limited settings, where access to clinical samples is often severely restricted. In this study, we encountered a similar limitation: the restricted availability of *B. suis* and *B. melitensis* isolates, as well as clinical tissue samples designated for experimental evaluation, posed a significant constraint on our analysis. This limitation arises primarily from the epidemiological situation in Canada, where porcine brucellosis (*B. suis*) and caprine/ovine brucellosis (*B. melitensis*) have not been reported in domestic livestock, and bovine brucellosis (*B. abortus*) has been eradicated from Canadian livestock since the 1980s (74). Consequently, the availability of local clinical tissue samples for *Brucella* isolation has continued to be significantly limited.

However, in contrast, *in silico* PCR analyses and validations are not subject to the same constraints. By leveraging extensive, publicly available WGS data, these computational approaches provide valuable preliminary insights and serve as a potential alternative to the challenges associated with obtaining clinical samples. Unfortunately, due to limitations of *in silico* PCR, the analysis was primarily restricted to qPCR formats using a single primer pair, with or without a single probe per target. Assays that employ multiple probes, such as hybridization or competitive probes, or multiple primer pairs for a single target, such as nested PCR, cannot be reliably evaluated within this framework. Furthermore, *in silico* PCR does not account for PCR kinetics or downstream signal behavior and therefore cannot be used to assess methods such as high-resolution melt (HRM) analysis. Consequently, as this study focuses on the development of conventional TaqMan-based qPCR assays using one primer-probe set per target, only a comparative *in silico* analysis for this specific qPCR format was conducted.

Our newly developed qPCR primer-probe sets targeting the *Brucella* genus, *B. abortus*, *B. melitensis,* and *B. suis* demonstrated outstanding *in silico* sensitivity and specificity, matching or surpassing the performance of previously published qPCR assays for the same targets (Table 2). Since this evaluation relied on assembled WGS data, a small number of apparent false negatives may arise from assembly/sequence errors, incomplete draft genomes, or ambiguous bases overlapping primer/probe binding sites. Therefore, these rare *in silico* misses should be interpreted cautiously and confirmed by inspecting the underlying sequences or wet-lab testing. Given these considerations, all primer-probe sets showing perfect or near-perfect performance (above 99.5% sensitivity and specificity), including primer-probe sets developed in this study and several previously published primer-probe sets for the *Brucella* genus, *B. melitensis,* and *B. suis*, represent strong candidates for further laboratory validation.

Overall, most genus-specific *Brucella* primer-probe sets targeting the *bcsp31* gene exhibited high *in silico* sensitivity and specificity, findings further supported by previous laboratory evaluations using bacterial cultures and clinical samples (43–47, 49). However, the most widely used qPCR targeting *bcsp31*, developed in 2004 (49), showed 85.2% *in silico* sensitivity (Table 2). Further investigation revealed a mismatch at the third nucleotide from the 3′ end of the reverse primer in a subset of 172 *B. melitensis* genome assemblies, leading to slightly lower sensitivity in the *in silico* PCR analysis (Table 2). Comparable variability in sensitivity has been reported in wet-lab evaluations using various types of samples, with sensitivities ranging from 49.1% to 93.9% (50, 75, 76). This mismatch is associated with a known SNP (360T>G), as confirmed by a previous study (77). Given its proximity to the primer’s 3′ ends, this SNP could theoretically affect qPCR efficiency (78). However, accurately predicting its precise impact remains challenging due to multiple factors, including polymerase fidelity, melting temperature, and buffer composition. Future studies are warranted to investigate the functional implications of this SNP on the performance of the *bcsp31*-based assay.

Similarly, *IS711*-based qPCR assays exhibited variable diagnostic sensitivity and specificity in laboratory evaluations (40). Consistently, in our *in silico* analysis, their sensitivity ranged from 74.3% to 86.4% (Table 2). This variability in *in silico* sensitivity is attributed to multiple mismatches across various species’ genome assemblies. Unfortunately, there are limited data on multiple SNPs within *IS711*, complicating our ability to distinguish between true SNPs and sequencing errors. Additional studies are essential to validate these SNPs and assess their impact on assay performance.

Other qPCR assays targeting *eryC*, *per,* or the 16S–23S intergenic transcribed spacer (ITS) region have demonstrated remarkable sensitivity and specificity in *in silico* analyses, closely mirroring their validated accuracy in wet laboratory environments (45, 57, 58). The *eryD* gene is an essential component of the *ery* operon, which mediates erythritol utilization. Homologs of *eryD* have been identified in *Brucella (Ochrobactrum*) species, with DNA sequences fully aligned to *eryD* in *Brucella* (79, 80). This likely explains the positive results observed in approximately 50% of *Brucella (Ochrobactrum*) genome assemblies during *in silico* screening, indicating cross-reactivity with closely related taxa. Additionally, the *virB4* gene encodes a core component of the Type IV secretion system. Some studies have reported that *Brucella* isolates lack *virB* (81), thereby reducing primer or probe target availability and decreasing *in silico* sensitivity. Confirmed SNPs in *virB4* have also been identified in certain *B. canis* isolates (82), and these sequence variants may further reduce *in silico* detection, depending on the location of mismatches within primer or probe binding sites.

Moreover, species-specific qPCR assays developed for the detection of *B. melitensis* (targeting the acetyl-CoA acetyltransferase gene) (61) and *B. suis* (targeting the *BS1330_II0657*) (64) achieved 100% accuracy in both laboratory evaluations and *in silico* analyses. In contrast, the IS711_alkB-based qPCR assay (49) designated for the specific detection of *B. abortus* exhibited inadequately low sensitivity, recorded at 58.6% during *in silico* analysis, which aligned with wet laboratory validation, revealing a sensitivity of merely 42.9% (51). Furthermore, the qPCR assays specifically designed to detect *B. melitensis* (targeting *BMEII0466*) and *B. suis* (targeting *BR0952*) could generate amplicons when utilizing genomic sequences from other *Brucella* species, including *B. neotomae* and *B. canis*, in the *in silico* analyses, an observation that was previously confirmed through wet laboratory validations (54). All these data presented herein underscore the comparable accuracy of *in silico* analysis relative to traditional experimental methodology.

However, *in silico* PCR analysis is highly dependent on the quality of genome assemblies and the accuracy of taxonomic assignments. In our study, we employed multiple approaches to revalidate taxonomic assignments (Table S5 and Fig. S2). Additionally, a completeness score threshold of 80% was used to assess genome assembly quality, as recommended by a previous study (37). Despite these measures, our evaluation revealed that even genome assemblies with high completeness scores but highly fragmented contigs negatively impacted the performance of *in silico* analyses for qPCR assays, as exemplified by our multiplex qPCR assay targeting *B. suis* (Table 2). This finding highlights the importance of not only considering completeness scores but also evaluating other quality metrics, such as contig number and assembly fragmentation, to improve the reliability of *in silico* PCR analyses and strengthen the validation of qPCR assays in future studies.

*In silico* PCR analysis is a powerful and efficient tool for assessing the sensitivity and specificity of primer and probe combinations, offering valuable insights during the early stages of assay development. It enables rapid screening and optimization, helping us to streamline the design process. However, it may not fully capture the complexities of PCR dynamics encountered in wet laboratory conditions, such as primer dimer formation, template structure, reaction kinetics, primer-template interactions, and the presence of inhibitors. Therefore, while *in silico* analysis provides a strong foundation, comprehensive laboratory validation remains essential for ensuring molecular assays’ robustness and diagnostic reliability.

Accordingly, our novel qPCR assays were evaluated and validated in the laboratory, demonstrating 100% sensitivity and specificity for each target using one of the largest genomic DNA validation panels to date (Table 2). These results confirm the high diagnostic accuracy of our qPCR assays and are in strong agreement with the predictions derived from the *in silico* PCR analysis (Fig. 2D and Table 2).

Beyond conventional TaqMan-based qPCR assays, other qPCR formats—such as hybridization-probe assays that employ two sequence-specific oligonucleotide probes, nested qPCR, and qPCR followed by high-resolution melting analysis—have also been developed for *Brucella* genus, species, or biovars level detection and have demonstrated high sensitivity and specificity in laboratory evaluations (40, 83, 84). Additionally, several published studies have evaluated commercial *Brucella* qPCR kits across different specimen types, mainly focusing on milk and serum, and indicate that assay performance is strongly matrix-dependent (85–88). In dairy matrices, commercial kit-based workflows have reported low limits of detection, ranging from 30 CFU/mL to 300 CFU/mL (88). In clinical serum samples, at least one commercial kit study reported 88% sensitivity and 100% specificity (85). Therefore, all these qPCR assays likewise warrant further laboratory validation across diverse panels and sample types to confirm their accuracy, reliability, and robustness.

The direct detection of *Brucella* DNA in clinical samples using PCR-based methods has become an increasingly recognized and valuable approach for diagnosing brucellosis (89). This method is preferred over traditional culture techniques due to its rapidity, sensitivity, and improved safety. However, PCR-based detection in clinical matrices, such as blood, serum, or tissue, presents significant challenges compared to pure bacterial cultures. These challenges arise primarily due to the high background of host DNA, abundant proteins, and various inhibitory substances that can significantly impair PCR efficiency. Therefore, preparation of tissue samples, including bacterial DNA recovery and PCR inhibitor removal, is a critical step in ensuring the success of subsequent qPCR assays (90), which are often underestimated. Previous studies have demonstrated that different commercial DNA extraction kits yield DNA of markedly varying quality and quantity, which can affect *Brucella* qPCR performance depending on the sample type (91–93). Nevertheless, most published comparisons focus on total DNA extraction and overlook the microbe-enrichment or host-depletion approach, which could substantially enhance molecular detection in specimens with high host content. In this study, we employed the Ultra-Deep Microbiome Prep kit with an improved workflow (42), which allowed us to increase the LOD of our qPCR assays by up to 400-fold (Fig. S1). To comply with biosafety requirements for *Brucella* handling and address the limited capacity and resources of the CL3 containment facility, a heat-inactivation process was implemented, allowing spiking and extraction experiments to be conducted safely in a CL2 laboratory. As a quality-control measure during heat inactivation, the release of “free” *B. suis* biovar 4 DNA into the supernatant after heat treatment was monitored, typically ranging from 0.147 to 11.2 ng/µL (mean 2.38 ng/µL, 95% CI: 1.39–3.38). When normalized to the estimated total DNA per sample, calculated from CFU-derived biomass estimates, the extracellular (“free”) DNA accounted for an average of 2.62% (95% CI: 1.77–3.48) of the total bacterial DNA. These findings indicate that most *Brucella* DNA remained cell-associated after inactivation. However, it should be noted that heat stress may increase cell envelope permeability, rendering cells more susceptible to lysis during subsequent extraction steps. Therefore, the spiking model assessed overall workflow performance rather than providing a direct, lysis-specific comparison of extraction kits, which constitutes a limitation of the study.

Recent investigations have confirmed that Rangiferine brucellosis, caused by *B. suis* biovar 4, remains endemic in Canada’s Arctic and sub-Arctic regions (94). This persistent presence underscores the urgent need for targeted surveillance of *B. suis* bv. 4, particularly in wildlife reservoirs such as caribou and muskoxen. Based on this epidemiological context, our current study focused on detecting *B. suis* biovar 4 in tissue samples using our qPCR assays. Sixteen culture-positive tissue samples were used in the current research, marking a promising step toward validating reliable molecular diagnostics. Expanding the sample size and fostering collaboration with laboratories worldwide in future studies will be essential for the complete validation of these assays, ultimately supporting their integration into routine diagnostic and surveillance programs.

### Conclusion

This study successfully developed two highly sensitive and specific real-time PCR assays for detecting *Brucella* species using large-scale whole-genome sequencing data mining. The newly designed multiplex assay facilitates the simultaneous identification of *B. abortus*, *B. melitensis*, and *B. suis*, while the genus-specific assay offers broad detection capabilities across various *Brucella* species. Both assays demonstrated exceptional sensitivity and specificity during extensive *in silico* analyses and laboratory evaluations, using genomic DNA extracted from bacterial cultures and clinical tissue samples. These findings emphasize the efficacy of these methods as reliable diagnostic tools for the swift and precise detection of *Brucella*, thereby enhancing disease surveillance and control efforts.

## Data Availability

Bacterial isolates are available from the corresponding author upon formal request. Access to these materials is subject to a Material Transfer Agreement and compliance with applicable biosafety regulations.

## SUPPLEMENTAL MATERIAL

File S1 spectrum.02974-25-s0001.docx Supplemental methods, Tables S1 to S5, and Fig. S1 to S3.

File S2 spectrum.02974-25-s0002.xlsx PubMed search results.

ASM does not own the copyrights to Supplemental Material that may be linked to, or accessed through, an article. The authors have granted ASM a non-exclusive, world-wide license to publish the Supplemental Material files. Please contact the corresponding author directly for reuse.
