# Supplementary material for: Two novel real-time PCR assays for Brucella detection: a species-specific multiplex and a genus-level singleplex developed via large-scale whole genome sequencing data analysis
Source: Microbiol Spectr. 2026 Mar 26;14(5):e02974-25. doi: 10.1128/spectrum.02974-25 (PMC13142026; doi:10.1128/spectrum.02974-25)
Supplement: File S1 — Supplemental methods, Tables S1 to S5, and Fig. S1 to S3. [file spectrum.02974-25-s0001.docx]

**Supplemental data for**

**Two Novel Real-Time PCR Assays for *Brucella* Detection: A Species-Specific Multiplex and a Genus-Level Singleplex Developed via Large-Scale Whole Genome Sequencing Data Analysis**

Emily Hoover^#^, John Chmara^#^, Marc-Olivier Duceppe, Philippe Charron, Dara Lloyd, Hongsheng Huang, Om Surujballi, Niroshan Thanthrigedon, Kristin Arnold, Jennifer Hazelwood, and Mingsong Kang*

Ottawa Laboratory Fallowfield, Canadian Food Inspection Agency, 3851 Fallowfield Road, Nepean, Ontario K2J 4S1, Canada.

**^#^**Contributed equally

^*^Address correspondence to Mingsong Kang, [mingsong.kang@inspection.gc.ca](mailto:mingsong.kang@inspection.gc.ca)

**Key words:** qPCR, *Brucella abortus*, *Brucella melitensis*, *Brucella suis*, whole genome sequences

**Methods**

**Re-evaluation of misidentified Brucella genome assemblies**

Putative misidentified *Brucella* genome assemblies were initially detected through phylogenetic analysis using in-house pipeline genome_comparator version 0.1 (<https://github.com/duceppemo/genome_comparator> ) and visualized by iTOL version 7.2(1). To confirm misclassification and refine species-level assignments, these assemblies were re-evaluated using four complementary methods: SNP-based Phylogenetic analysis, PubMLST(2), GTDB-Tk(3), and in-silico PCR.

SNP-based phylogenetic analysis**:** Reference genomes of *Brucella* species, along with misidentified genome assemblies, were used to construct a SNP-based phylogenetic tree, following the approach described in the previous study(4).

PubMLST: Each genome assembly was analyzed via the “Identify species” online tool available at PubMLST (<https://pubmlst.org/bigsdb?db=pubmlst_rmlst_seqdef_kiosk>).

GTDB-Tk: The Genome Taxonomy Database Toolkit (GTDB-Tk v2.3.2, reference data release r220) was employed to assign standardized taxonomic labels to the suspect assemblies. Analyses were performed using default parameters.

In-silico PCR: Primer pairs developed in this study (Table 1), targeting genus- and species-specific regions of *Brucella*, were used to simulate PCR amplification with in_silico_PCR v0.5 (<https://github.com/duceppemo/insilicoPCR> ) with 3 mismatches tolerance.

**Average nucleotide identity analysis of *Brucella* genome assemblies**

A total of 1,163 *Brucella* genome assemblies (1,160 classical *Brucella* species and 3 *B. inopinata*) retrieved from the NCBI RefSeq database (as of March 2025) were subjected to average nucleotide identity (ANI) analysis. ANI was calculated between each assembly and reference genomes of known *Brucella* species using **FastANI** v1.34 (5). Results were visualized using the Python v3.10.12 seaborn module v0.13.2.

**Supplemental Tables**

**Table S1.** Bacterial isolates tested to assess the sensitivity and specificity of qPCR assays

|  | **Species/genus** | **Number** |
| --- | --- | --- |
| **Classical *Brucella***  **(235)** | *B. abortus* | 170 |
|  | *B. suis* | 38 |
|  | *B. melitensis* | 5 |
|  | *B. ovis* | 3 |
|  | *B. canis* | 12 |
|  | *B. cetaeae* | 5 |
|  | *B. microti* | 1 |
|  | *B. neotomae* | 1 |
| **Non-classical *Brucella or Non Brucella***  **(192)** | *E. coli* | 21 |
|  | *Enterobacter* | 5 |
|  | *Listeria* | 4 |
|  | *Salmonella* | 142 |
|  | *Klebsiella* | 4 |
|  | *Staphylococcus* | 2 |
|  | *Yersinia enterocolitica* ATCC 55075 | 1 |
|  | *Brucella (Ochrobactrum)* | 1 |
|  | *Aeromonas* | 2 |
|  | *Alcaligenes faecalis* | 1 |
|  | *Campylobacter* | 1 |
|  | *Citrobacter freundii* | 2 |
|  | *Serratia fonticola* | 2 |
|  | *Raoultella* | 2 |
|  | *Proteus vulganis* | 1 |
|  | *Providencia rettgeri* | 1 |

**Table S2.** Genome assemblies used for in-silico evaluation

|  | **Species** | **Number^$^** |
| --- | --- | --- |
| **Classical *Brucella***  **(1160)** | *B.abortus* | 360 |
|  | *B. suis* | 81+4^@^ |
|  | *B.melitensis* | 642 |
|  | *B. canis* | 29 |
|  | *B.ceti* | 20 |
|  | *B. ovis* | 17 |
|  | *B. neotomae* | 6 |
|  | *B.microti* | 1 |
| **Non-Classical *Brucella* or Non*-Brucella***  **(194)** | *Pseudochrobactrum* | 22 |
|  | *Paenochrobactrum* | 7 |
|  | *Brucella (Ochrobactrum)* | 159 |
|  | *Falsochrobactrum* | 6 |

**^$^**Genome assemblies were downloaded from The NCBI RefSeq database in March 2025

**^@^**There are four genome assemblies of *B. suis* bv. 5

**Table S3.** Summary of tissue samples utilized for qPCR assay evaluation

| **Sample type** | **Tissue** |
| --- | --- |
| Negative control sample | Porcine lymphoid tissues include spleens and pooled LNs from the head, neck, and body |
|  |  |
| Negative clinical sample^@^  (9) | Joint |
|  | Joint capsule from left hind leg |
|  | Leg abscess within the muscle from the left hind leg |
|  | Lung |
|  | Mandibular LNs, Parotid LNs, Medial/Lateral Retropharyngeal LNs, |
|  | Cervical caudal LNs, Caudal Mediastinal LNs |
|  | Spleen |
|  | Superficial cervical (Prescapular LNs), Mammary (Superficial Inguinal LNs), Subiliac (Prefemoral LNs), Medial iliac (Internal Iliac adjacent to spine) |
|  | Mandibular LNs, Parotid LNs, Medial/Lateral Retropharyngeal LNs, |
|  |  |
| Positive clinical sample^@^  (16) | Joint capsule |
|  | Joint capsule and testicle |
|  | Lymph Node (unidentified) Muscle and Abscess |
|  | Unidentified Lymph Node |
|  | Joint tissue |
|  | Muscle abscess |
|  | Lesion from right hind leg in the muscle of the thigh |
|  | Joint capsule and a swab of a granuloma |
|  | Abscess from the hind quarter between dew claws |
|  | White nodule on kidney |
|  | Abnormal cystic structure from unknown location |
|  | Cystic mass over carpal joint |
|  | Swelling on carpal joint |
|  | Swelling on carpal joint |
|  | Hygroma capsule near carpal joint |
|  | Joint tissue |

LN: lymph node

^@^ Samples collected from wildlife animals

**Table S4.** *In silico* PCR results for two over- fragmented *B. suis* assemblies removed from data analysis

| **accession** | **qseqid** | **primerprobe** | **mismatch** | **gaps** | **length** | **qstart** | **qend** | **Size**  **(bp)** |
| --- | --- | --- | --- | --- | --- | --- | --- | --- |
| GCF_000292005.1 | NZ_ALOK02000260.1 | BS-P | 0 | 0 | 24 | 539 | 562 | 577 |
|  | NZ_ALOK02000260.1 | BS-R | 0 | 0 | 20 | 497 | 516 |  |
|  | NZ_ALOK02000046.1 | BS-F | 0 | 0 | 21 | 1 | 21 | 20977 |
|  |  |  |  |  |  |  |  |  |
| GCF_000292105.1 | NZ_ALON02000226.1 | BS-P | 0 | 0 | 24 | 16 | 39 | 577 |
|  | NZ_ALON02000226.1 | BS-R | 0 | 0 | 20 | 62 | 81 |  |
|  | NZ_ALON02000030.1 | BS-F | 0 | 0 | 21 | 1 | 21 | 106839 |

**Table S5.** Misidentification of *Brucella* genome assemblies in the *RefSeq* database

| **species identification (RefSeq)** | **PubMLST** | **GTDB-TK** | **phylogeny** | ***In_silico* PCR** |
| --- | --- | --- | --- | --- |
| *B. suis* S2-30  (GCF_000292125.1) | *B.melitensis* (100%) | *B.melitensis* | *B.melitensis* | *B.melitensis* |
| *B. melitensis* HN20190002 (GCF_009823695.1) | *B.suis* (100%) | *B.melitensis* | *B.suis* | *B.suis* |
| *B. inopinata* BO1 (GCF_000662015.2) | *B.canis* (100%) | *B.melitensis* | *B.canis* | *n.a.* |
| *B. melitensis* S66 (GCF_000250775.1) | *B.suis* (100%) | *B.melitensis* | *B.suis* | *B.suis* |
| *B. melitensis* 16M13W (GCF_000250835.1) | *B.abortus* (100%) | *B.melitensis* | *B.abortus* | *B.abortus* |
| *B. abortus* BCB027  (GCF_000292145.1) | *B. melitensis* (88%)  *B.abortus* (11%) | *B.melitensis* | *B. melitensis* | *B. melitensis* |

n.a.: not applicable

**Supplemental Figures**


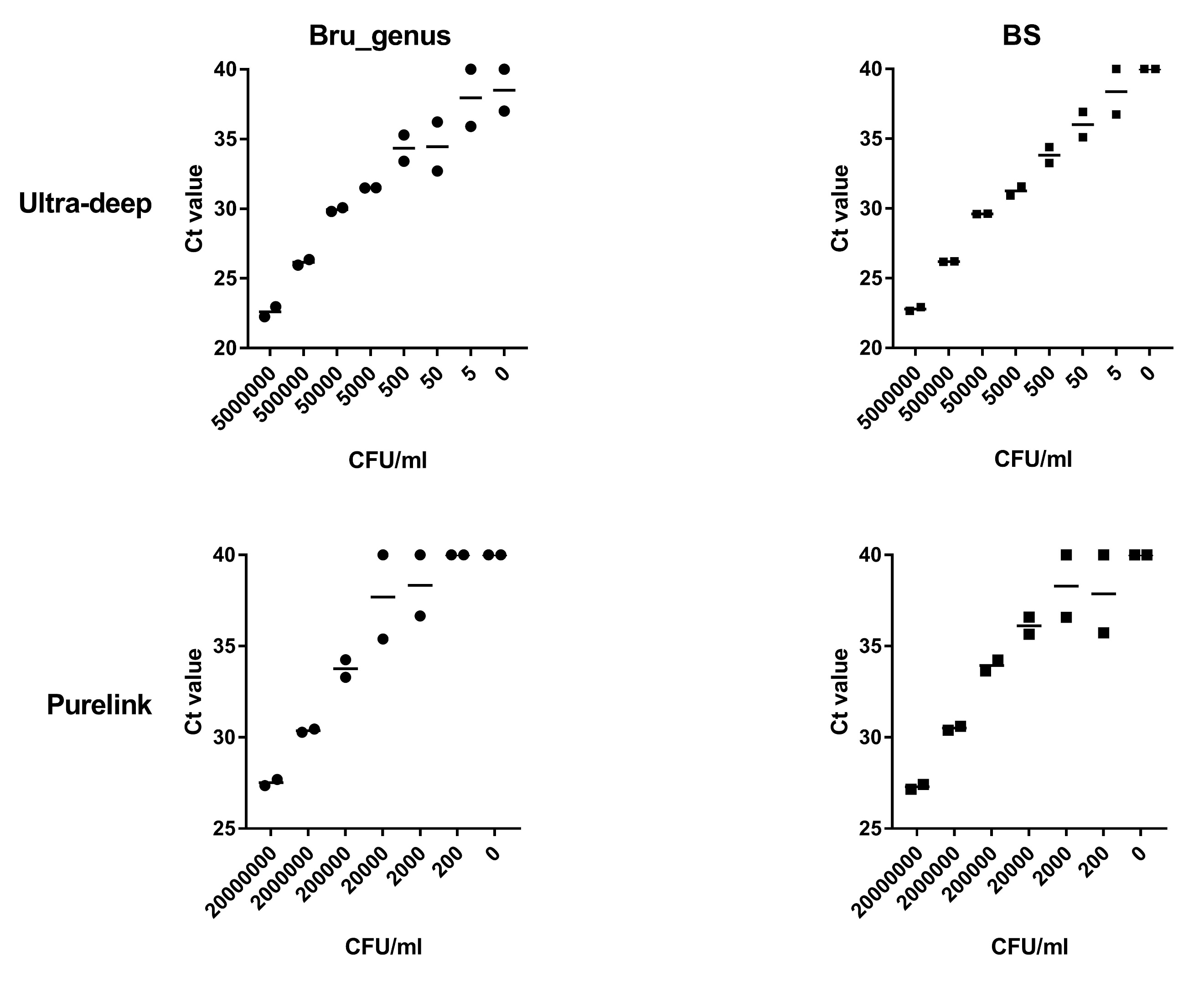


**Figure S1.** Comparative study on the performance of bacterial enrichment between the Ultra-Deep Microbiome Prep Kit and the Purlink Genomic DNA Kit. The efficiency of each method was assessed using two qPCR assays: Bru_genus, a genus-specific *Brucella* qPCR assay targeting classical *Brucella* species, and BS, a multiplex qPCR assay targeting *B. suis*. Ct values recorded as "Undetermined" in qPCR assays were set to 40 for data analysis.

**A**


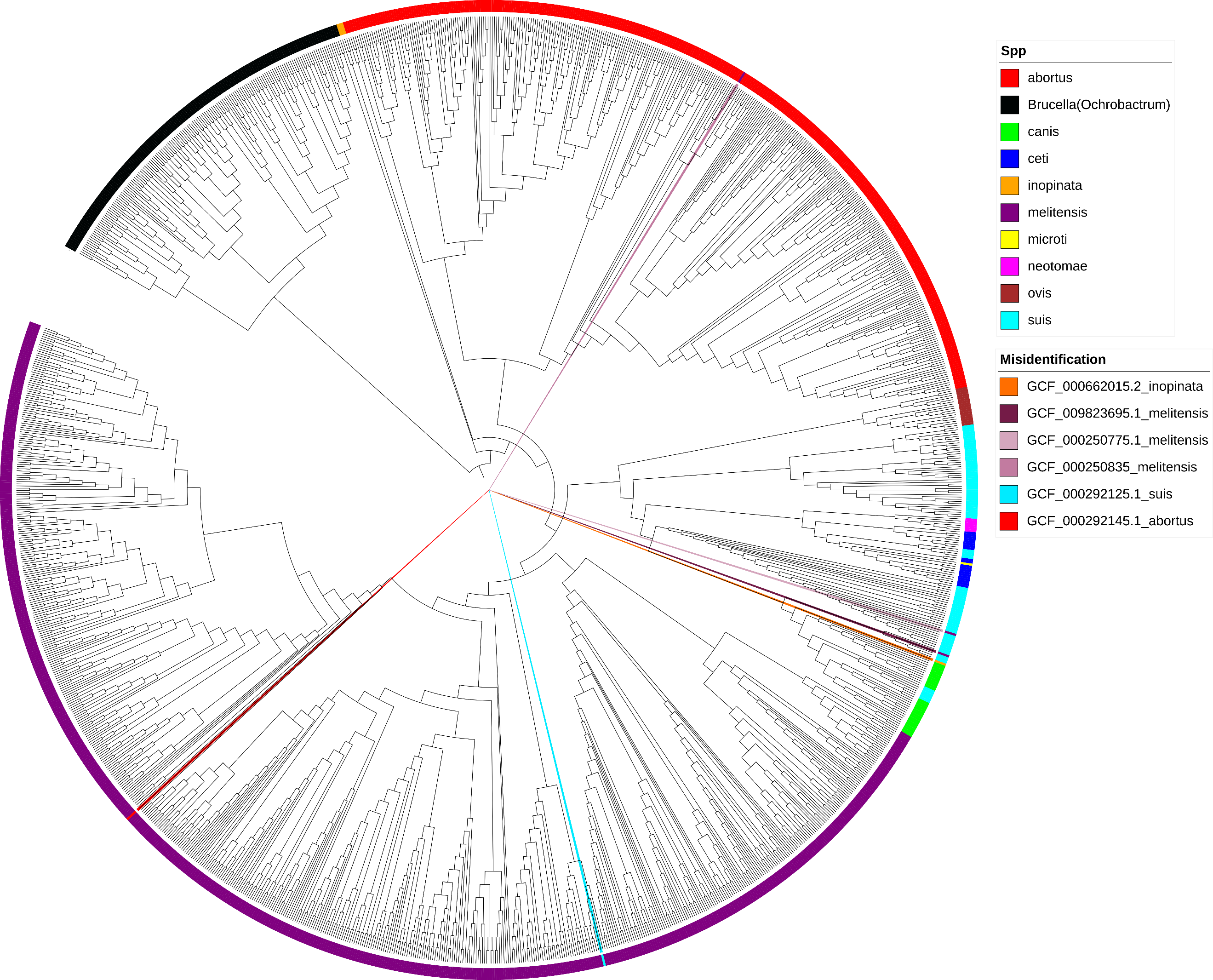


**B**

**
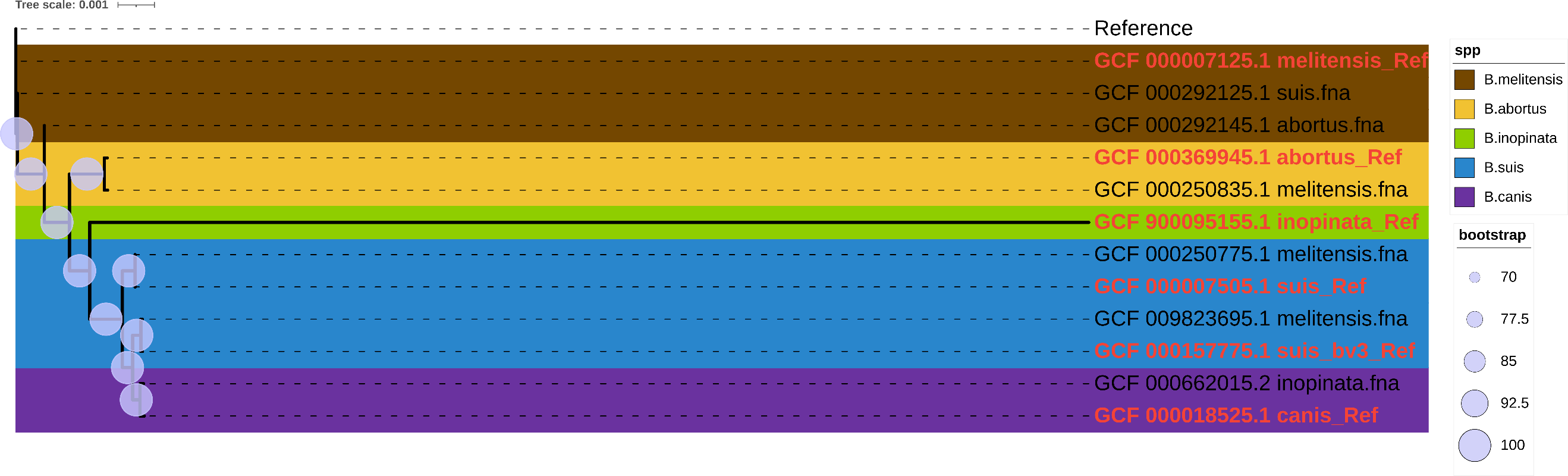
**

**Figure S2.** Phylogenetic analysis of *Brucella* genome assemblies. (A) Mash distance-based phylogenetic tree constructed from 1,328 *Brucella* genome assemblies (as of March 2025). Branch lengths were not considered. Color strips indicate different *Brucella* species, and shaded regions highlight misidentified assemblies. (B) SNP-based phylogenetic tree. Color ranges represent distinct *Brucella* species, and red text denotes reference genomes.

`


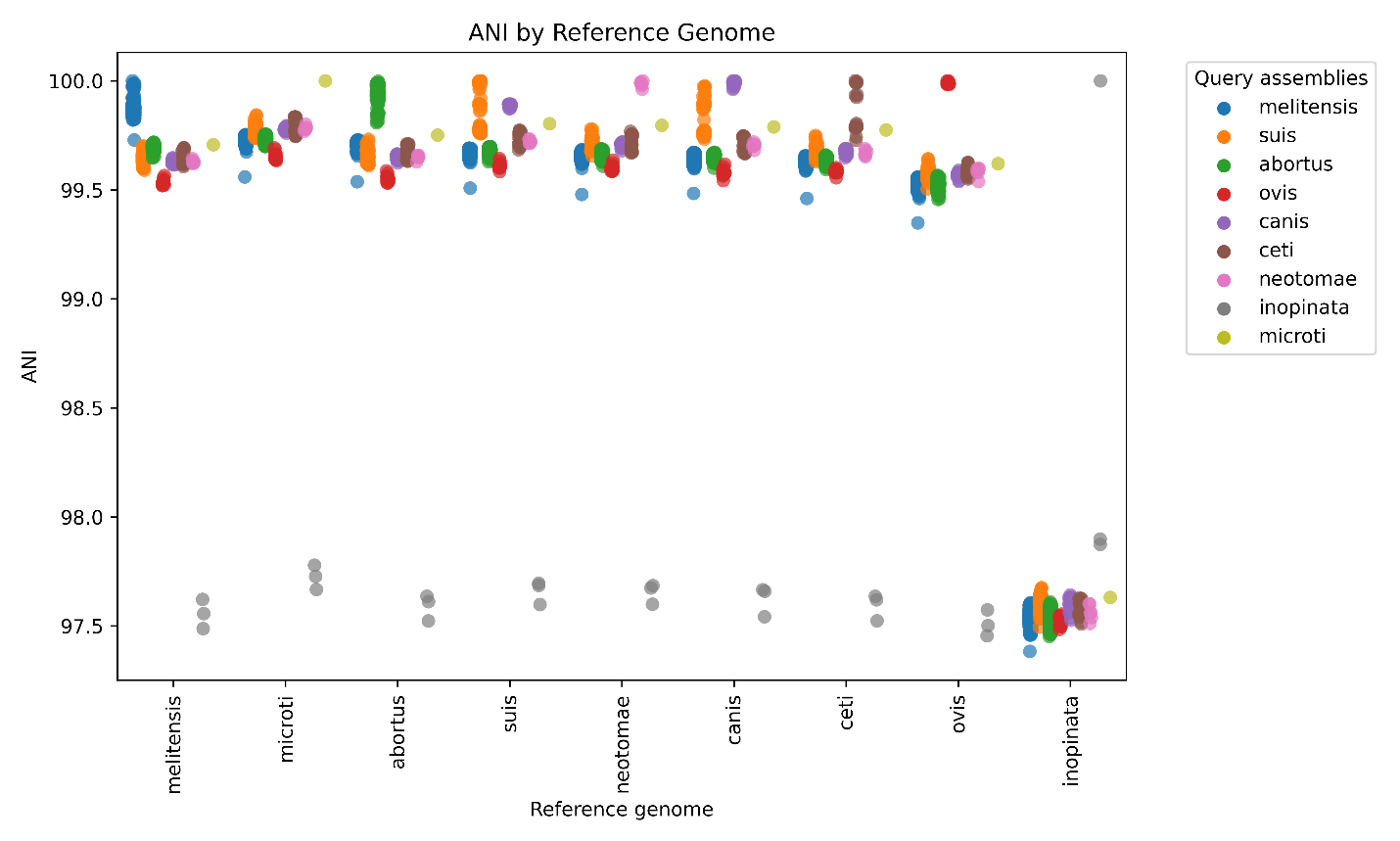


**Figure S3.** Average Nucleotide Identity (ANI) values between query and reference genomes of *Brucella*. Each dot, color-coded by the species of the query genomes, represents the ANI between a query genome and a reference genome. The following *Brucella* reference genomes were used: *B. melitensis* (GCF_000007125.1), *B. suis* (GCF_000007505.1), *B. canis* (GCF_000018525.1), *B. ovis* (GCF_000016845.1), *B. microti* (GCF_90280745.1), *B. inopinata* (GCF_900095155.1), *B. abortus* (GCF_000369945.1), *B. neotomae* (GCF_900446125.1), and *B. ceti* (GCF_047324105.1).

**References**

1. Letunic I, Bork P. 2024. Interactive Tree of Life (iTOL) v6: recent updates to the phylogenetic tree display and annotation tool. Nucleic Acids Res 52:W78-W82.

2. Jolley KA, Bray JE, Maiden MCJ. 2017. A RESTful application programming interface for the PubMLST molecular typing and genome databases. Database (Oxford) 2017.

3. Chaumeil PA, Mussig AJ, Hugenholtz P, Parks DH. 2022. GTDB-Tk v2: memory friendly classification with the genome taxonomy database. Bioinformatics 38:5315-5316.

4. Charron P, Gao R, Chmara J, Hoover E, Nadin-Davis S, Chauvin D, Hazelwood J, Makondo K, Duceppe MO, Kang M. 2023. Influence of genomic variations on glanders serodiagnostic antigens using integrative genomic and transcriptomic approaches. Front Vet Sci 10:1217135.

5. Jain C, Rodriguez RL, Phillippy AM, Konstantinidis KT, Aluru S. 2018. High throughput ANI analysis of 90K prokaryotic genomes reveals clear species boundaries. Nat Commun 9:5114.
